# Palbociclib Induces Senescence in Melanoma and Breast Cancer Cells and Leads to Additive Growth Arrest in Combination With Irradiation

**DOI:** 10.3389/fonc.2021.740002

**Published:** 2021-10-13

**Authors:** Tina Jost, Lucie Heinzerling, Rainer Fietkau, Markus Hecht, Luitpold V. Distel

**Affiliations:** ^1^ Department of Radiation Oncology, University Hospital of Erlangen, Friedrich-Alexander University Erlangen-Nürnberg, Erlangen, Germany; ^2^ Comprehensive Cancer Center Erlangen-EMN, Erlangen, Germany; ^3^ Department of Dermatology, University Hospital of Munich, Ludwig-Maximilian University Munich (LMU), Munich, Germany

**Keywords:** senescence, kinase inhibitor, radiotherapy, palbociclib, breast cancer, melanoma

## Abstract

**Introduction:**

Several kinase inhibitors (KI) bear the potential to act as radiosensitizers. Little is known of the radiosensitizing effects of a wide range of other KI like palbociclib, which is approved in ER+/HER2- metastatic breast cancer.

**Method:**

In our study, we used healthy donor fibroblasts and breast cancer and skin cancer cells to investigate the influence of a concomitant KI + radiation therapy. Cell death and cell cycle distribution were studied by flow cytometry after Annexin-V/7-AAD and Hoechst staining. Cellular growth arrest was studied in colony-forming assays. Furthermore, we used C12-FDG staining (senescence) and mRNA expression analysis (qPCR) to clarify cellular mechanisms.

**Results:**

The CDK4/6 inhibitor palbociclib induced a cell cycle arrest in the G0/G1 phase. Cellular toxicity (cell death) was only slightly increased by palbociclib and not enhanced by additional radiotherapy. As the main outcome of the colony formation assays, we found that cellular growth arrest was induced by palbociclib and improved by radiotherapy in an additive manner. Noticeably, palbociclib treatment clearly induced senescence not only in breast cancer and partly in melanoma cells, but also in healthy fibroblasts. According to these findings, the downregulation of senescence-related FOXM1 might be an involved mechanism of the senescence-induction potential of palbociclib.

**Conclusion:**

The effect on cellular growth arrest of palbociclib and radiotherapy is additive. Palbociclib induces permanent G0/G1 cell cycle arrest by inducing senescence in fibroblasts, breast cancer, and melanoma cells. Direct cell death induction is only a minor secondary mechanism of action. Concomitant KI and radiotherapy is a strategy worth studying in clinical trials.

**Graphical Abstract:**
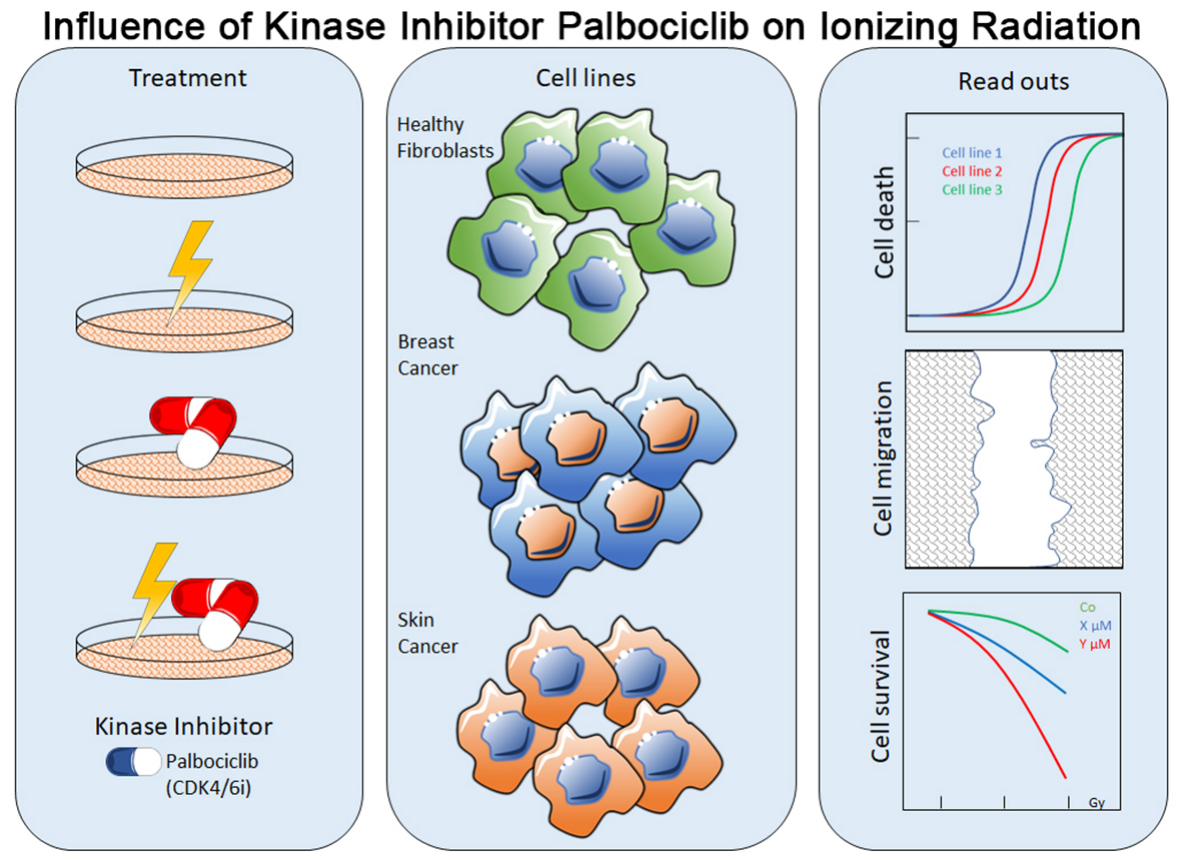


## Introduction

Female breast cancer is the most commonly diagnosed cancer. Citing the European Cancer Information System (ECIS), over 355,000 women in the EU-27 are estimated to be diagnosed with breast cancer in 2020 (1). This represents 13.3% of all cancer diagnoses. Between 2015 and 2017, approximately 13% of all female patients were treated with palbociclib (2). Palbociclib is a kinase inhibitor (KI) that blocks the cell cycle by inhibiting the phosphorylation of the Rb protein and was approved by the European Medicines Agency (EMA) in 2016 for ER+ and HER2- metastatic breast cancer (3). In essence, kinase inhibitors targeting different proteins in important cellular pathways are gaining more and more attention in the treatment of cancer patients. Moreover, the question of combining different therapy options like concomitant radiotherapy (RT) with focus on radiosensitization arises (4), especially in the metastatic situation, since metastases are commonly treated with irradiation (5).

Previous studies showed that kinase inhibitors are able to act as a radiosensitizer and therefore can enhance tumor control (6, 7), but have side effects on healthy tissue too. Radiosensitizing potential was found for BRaf V600E inhibitors vemurafenib and dabrafenib (8, 9). Consequently, a hold of KI treatment more than 3 days before and after fractionated RT and hold of more than 1 day pre- and post-stereotactic radio surgery (SRS) are recommended by the Eastern Cooperative Oncology Group (ECOG) (10). Nevertheless, current data indicate an enhancement of local tumor control when KI therapy is combined with intracranial stereotactic RT without an increase of radionecrosis (11).

To enhance the treatment of cancer patients, a combination of KI and IR could be beneficial. However, the possible radiosensitizing effects of the cyclin-dependent kinase 4 (CDK4) and cyclin-dependent kinase 6 (CDK6) inhibitor palbociclib are not limited to tumor cells alone, but can also affect healthy cells. Additionally, healthy tissue like the skin is always affected during irradiation. Keeping these mechanisms of action in mind, our study focused on cellular response of tumor and healthy donor cells regarding cell death, cell cycle regulation, and senescence. Just few is known on the ability of palbociclib to trigger senescence. Nevertheless, this type of replicative G1-arrest is a main cellular outcome of radiation, since a fraction of the induced highly complex DNA damage cannot be repaired (12). Remarkably, the dependency of CDK4/6 in not limited to breast cancer alone, but to a wide range of entities like hepatocellular carcinoma, bronchial carcinoma, and head and neck squamous cell carcinoma (13–15). Increased CDK4 activity was also found in melanomas (16). This supports our intention of studying the effects of concomitant kinase inhibitor and RT not only in breast cancer cells, but also in skin cancer cells.

## Materials and Methods

### Cell Lines and Kinase Inhibitor

Human skin fibroblasts SBLF7 and SBLF9 derived from different healthy donors; melanoma cells LIWE, HV18MK, ICNI, RERO, ARPA, and ANST derived from different malign melanoma patients; and breast cancer cell lines MDA-MB-231 (TNBC) and MCF-7 (ER+, PR+) were used. MDA-MB-231 and MCF-7 were purchased by CLS cell lines service (Eppelheim, Germany). Primary human melanoma cells (from primary tumors) were collected in the Department of Dermatology of the Universitätsklinikum Erlangen following approval by the institutional review board (Ethic approval no. 204 17 BC). Single-cell suspensions were generated by digesting tissue samples with collagenase (Sigma Aldrich, München, Germany), hyaluronidase (Sigma Aldrich, München, Germany), and DNase (Roche, Mannheim, Germany) (17). The primary human fibroblasts SBLF7 and SBLF9 were isolated *via* skin biopsy of the cutis and subcutis after local anesthesia as described previously (18). Briefly, each biopsy was dissected in small pieces, placed in tissue culture flasks, and each covered with a drop of F-12 medium (Gibco, Waltham, USA) supplemented with 40% fetal bovine serum (FBS) (Merck, Darmstadt, Germany). After the skin pieces had attached to the culture flasks and the first fibroblasts had grown out, they were covered with F-12 medium supplemented with 12% FBS, 2% non-essential amino acids (NEA) (Merck, Darmstadt, Germany), and 1% penicillin/streptomycin (Gibco, Waltham, USA). When the primary cells were approximately 80% confluent, they were detached with 0.5% Trypsin (Gibco, Waltham, USA) and further cultured in the medium mentioned above. For continuous cell culture, fibroblasts were cultured in F-12 medium, supplemented with 15% FBS, 2% NEA, and 1% penicillin/streptomycin. Melanoma cells were cultured in RPMI-1640 (Sigma Aldrich, München, Germany), supplemented with 20% FBS (Merck, Darmstadt, Germany), 1% NEA (Merck, Darmstadt, Germany), 1% Pyruvate solution (Gibco, Waltham, USA), 1% L-Glutamine (Merck, Darmstadt, Germany), 1% HEPES (Merck, Darmstadt, Germany), and 0.05% Gentamicin (Merck, Darmstadt, Germany). Breast cancer cell lines were cultured in DMEM (PAN biotech, Aidenbach, Germany), supplemented with 10% FBS and 1% penicillin/streptomycin. All cells were incubated at 37°C in a humidified 5% CO_2_ atmosphere. Cells were cultured 50 passages maximum. Palbociclib isethionate (MW 573.7 g/mol) (Selleck Chemicals LLC, Huston, USA) was prepared as stock solution in aqua bidest and stored at −80°C with a concentration of 1 mmol/L. The drug was diluted for experiments in dimethyl sulfoxide (DMSO) (Roth, Karlsruhe, Germany). Required aliquots were thawed freshly prior to each experiment.

### Cell Death Analysis—FACS

Cells were washed with PBS (Sigma Aldrich, St. Louis, USA) and incubated with Trypsin/EDTA (Gibco, Waltham, USA) to detach the cells from cell culture flask to prepare a single-cell suspension. To reach a confluence of 50% up to 80% in 24 h up to 72 h, cells were seeded in an appropriate concentration. To reduce analytical interference and avoid an artificial increase of possible effects of our treatment through stimulation of cell proliferation (19–21), medium was exchanged for the experiments by serum-reduced cell culture medium (2% FBS) including 10 µl of various palbociclib concentrations. Previously, we checked if serum starvation influences our cell death analysis (data shown in **Supplementary Figure S1**). We diluted palbociclib in a certain manner so that we always had to add 10 µl of dilution per 1 µM and 2 µM and 10 µl of pure DMSO (Roth, Karlsruhe, Germany) to the control. Finally, we had a DMSO concentration of less than or equal to 1%. We compared this DMSO concentration with controls and found no effect (data shown in **Supplementary Figure S2**), which is similar to other findings (22).

Cells were incubated in the presence of the inhibitor for 48 h at 37°C. Additionally, half of the cells were irradiated with 2 Gy ionizing radiation (IR) by an ISOVOLT Titan X-ray generator (GE, Ahrensburg, Germany) 3 h after addition of inhibitor. Supernatant was collated and treated cells were harvested by trypsination. After washing, cells were resuspended in 200 µl of Ringer solution and stained with Annexin V-APC (BD, Heidelberg, Germany) and 7-amino-actinomycin D (BD, Heidelberg, Germany) for 30 min on ice. To analyze apoptotic and necrotic cells using flow cytometry (Cytoflex, Beckman Coulter, Brea, USA), cell suspension was transferred to 96-well plates. Excitation at 660/10 nm (Annexin V-APC) and 546 nm (7-AAD) was used to measure stained cells. Double-negative (Ann-7AAD-) cells were defined as alive, Ann+7AAD- cells as apoptotic, and Ann+7AAD+ cells as necrotic.

### Cell Cycle Analysis—FACS

After harvesting, cells were fixed in 10 ml of 70% ethanol (Roth, Karlsruhe, Germany) and 1 ml of serum-reduced cell culture medium for a minimum of 12 h at + 4°C and then stained with Hoechst 33342 (Invitrogen, Eugene, USA) for 60 min on ice. Because Hoechst 33342 is highly DNA-specific (preferentially binds to A-T base pairs), no RNA-digest is needed (Technical data sheet, BD Pharmingen). Cells were analyzed in the Cytoflex flow cytometer. In general, cells need approximately 24 h to go through cell cycle (Cooper, The cell—a molecular approach, 2nd Edition, 2000). To clearly identify any changes in cell cycle distribution, like G0/G1 or G2/M arrest, treatment of 48 h could be advisable. To test whether 24 h or 48 h of treatment should be done, we tested three cell lines previously (data shown in **Supplementary Figure S3**) and performed all experiments later on with 48 h of treatment.

### Colony-Forming Assay

Cells were seeded in six-well plates with a density of 100–2,000 cells per well. Cells were treated with different concentrations of inhibitor and irradiated after 3 h with a 0- or 2-Gy dose. After another incubation phase of 24 h, medium was exchanged by fresh standard medium without any drug and the inhibitor was washed out. Plates were incubated for 10 to 14 days until colonies of minimum 50 cells were developed. Colonies were stained with methylene blue (Sigma Aldrich, München, Germany) for 30 min at room temperature and counted when dry.

### C12-FDG Staining—Senescence

To investigate evolving senescence during KI or irradiation treatment, we seeded cells at low confluence in cell culture flasks. After 24 h of settlement, cells were treated with either kinase inhibitor, irradiation, or combination of both. As a control, cells were treated with DMSO only. On day 6 after treatment, medium was exchanged including DMSO or kinase inhibitor again. After 10 days of treatment, cells were collected and stained as previously published (23). Briefly, cells were treated with 100 nM Bafilomycin A1 (Merck, Darmstadt, Germany) for 30 min (37°C). Afterwards, Hoechst dye was added for another 30 min (37°C) and finally cells were treated with C12-FDG for 60 min (37°C). After centrifugation cells were resuspended in Ringer solution and stained with Annexin V-APC and 7-AAD (BD, Heidelberg, Germany) for 30 min on ice. Finally, C12-FDG positive cells were measured using flow cytometry (Cytoflex S, Beckman Coulter, Brea, CA, USA).

### Quantitative PCR—mRNA Expression Analysis

For analysis of mRNA expression after KI treatment, irradiation or the combination of both qRT-PCR was used as described previously (24). Briefly, cells were seeded in six-well-plates, treated with 2 µM palbociclib, 2 Gy dose, or the combination of both (2 µM + 2 Gy) and harvested 48 h after treatment. Cells were harvested by lysis with Trizol (peqlab, Darmstadt, Germany) and frozen (−80°C) immediately. RNA isolation was done with phenol-chloroform extraction and isolated RNA was frozen again. Genomic DNA was digested with DNase I Kit (Thermo Fisher Scientific, Waltham, USA) at 37°C for 30 min (Thermocycler, BIO-RAD, Hercules, CA, USA). RNA was transferred into cDNA *via* High-capacity RT kit (Thermo Fisher Scientific, Waltham, USA) and cDNA was diluted with water and Yellow dye (Thermo Fisher Scientific, Waltham, MA, USA). qRT-PCR was run using DyNAmo ColorFlash SYBR Green qPCR Kit (Thermo Fisher Scientific, Waltham, MA, USA). Bio-Rad primers (**Tables 1**, **2**, Bio-Rad Laboratories, Inc., Hercules, CA, USA) were used according to the manufacturer’s instructions. Two technical replicates (duplicate wells) from one RNA/cDNA preparation (one biological sample) were measured.

**Table 1:** Primer for target genes.

Gene	Primer	Unique Assay ID	Application
cyclin D1	CCND1	qHsaCID0013833	Inhibits autophagy
forkhead box M1	FOXM1	qHsaCED0004022	Inhibits senescence
myristoylated alanine-rich protein kinase C substrate	MARCKKS	qHsaCED0045667	Promotes migration, invasion
p16 (Cyclin-dependent kinase inhibitor 2A)	CDKN2A	qHsaCED0056722	CKD4 inhibitor
regulatory associated protein of MTOR, complex 1	RPTOR	qHsaCID0016865	Promotes autophagy
RPTOR independent companion of MTOR, complex 2	RICTOR	qHsaCID0007506	Promotes autophagy
SMAD family member 3	SMAD3	qHsaCID0008503	Inhibits G1/S-progression
v-myc myelocytomatosis viral oncogene homolog (avian)	MYC	qHsaCID0012921	Inhibits apoptosis

**Table 2:** Primer for housekeeper genes.

Gene	Primer	Unique Assay ID	Application
hydroxymethylbilane synthase	HMBS	qHsaCID0038839	Housekeeper
ribosomal protein L30	RPL30	qHsaCED0038096	Housekeeper
ubiquitin C	UBC	qHsaCED0023867	Housekeeper

### Statistical Analysis

GraphPad prism 9 software (San Diego, CA, USA) was used to perform statistical analysis. Non-parametric, unpaired one-tailed Mann–Whitney *U* test was used to analyze data, based on the minimum number of *n* = 3 experiments. *p*-value ≤ 0.05 was determined as significant. Graphs were also generated using GraphPad Prism 9 software.

### Ethics Approval and Consent to Participate

Ethical approval was obtained in the Department of Dermatology, Universitätsklinikum Erlangen following approval by the institutional review board (Ethik-Kommission der Friedrich-Alexander-Universität Erlangen-Nürnberg, approval No. 204_17 Bc). The patients provided written informed consent.

## Results

### Cell Death Is a Minor Way of Action in Palbociclib Treatment

**Figure 1:**
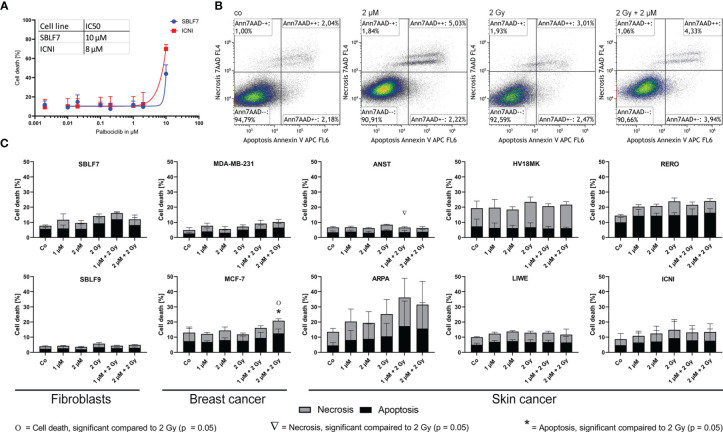
Cell death analysis of different non-malign and malign cell lines. **(A)** Dose escalation study of the kinase inhibitor palbociclib in healthy fibroblasts and skin cancer cells [dose–response curve fitting by non-linear regression of log(agonist) vs. response based on Graph Pad Prism]. **(B)** Gating strategy of the Annexin-7AAD (cell death) staining of skin cancer cells RERO after KI treatment, irradiation, and combi therapy. **(C)** Necrotic and apoptotic populations in 10 different cell lines (healthy fibroblast SBLF7 and SBLF9, breast cancer MDA-MB-231 and MCF-7, and skin cancer ANST, ARPA, HV18MK, LIWE, RERO, and ICNI cells) after 48 h of treatment. Each value represents mean ± SD (*n* = 3). Significance was determined by one-tailed Mann–Whitney *U* test Ο/Δ/* *p* ≤ 0.05.

To analyze the influence of kinase inhibitor palbociclib on RT, we investigated two fibroblast cell lines, as healthy controls, and two breast cancer and six skin cancer cell lines. First of all, cell death was measured by Annexin-7AAD staining using flow cytometry (**Figure 1**). The first dose escalation study (**Figure 1A**) showed IC50 values of 8 µM for the skin cancer cell line ICNI and 10 µM for the healthy fibroblasts SBLF7. Concerning the pharmacokinetics of palbociclib, we proceeded with physiologically achievable concentrations of 1 µM and 2 µM (25). Annexin-7AAD- cells were defined as “alive”, Annexin+7AAD- cells as “apoptotic”, and Annexin+7AAD+ cells as “necrotic” (**Figure 1B**).

Regarding our clinical context of the radiation oncology, we compared irradiation (IR) to the combination therapy (KI + IR), since KI can act as radiosensitizer and enhance the effect of IR in healthy tissue (side effects) and in tumor tissue leading to improve tumor control. The clinically most frequently used single dose of 2 Gy was chosen. Healthy fibroblasts did not show significant changes in apoptosis and necrosis (**Figure 1C**). Breast cancer cell line MCF-7 showed significant increase of apoptosis and total cell death after combination therapy with 2 µM and 2 Gy IR respectively (*p* = 0.05). The group of our tested skin cancer cell lines seemed to show diverse behavior, regarding strong tendencies, but not significant response in ARPA, and slight treatment-related response in RERO. HV18MK, ICNI, and LIWE did not respond in a treatment-related manner. Interestingly, in the skin cancer cell line ANST, necrosis was significantly reduced in combination of KI and IR (*p* = 0.05).

### Palbociclib and IR Influence Clonogenicity and Cell Survival in an Additive Manner

**Figure 2:**
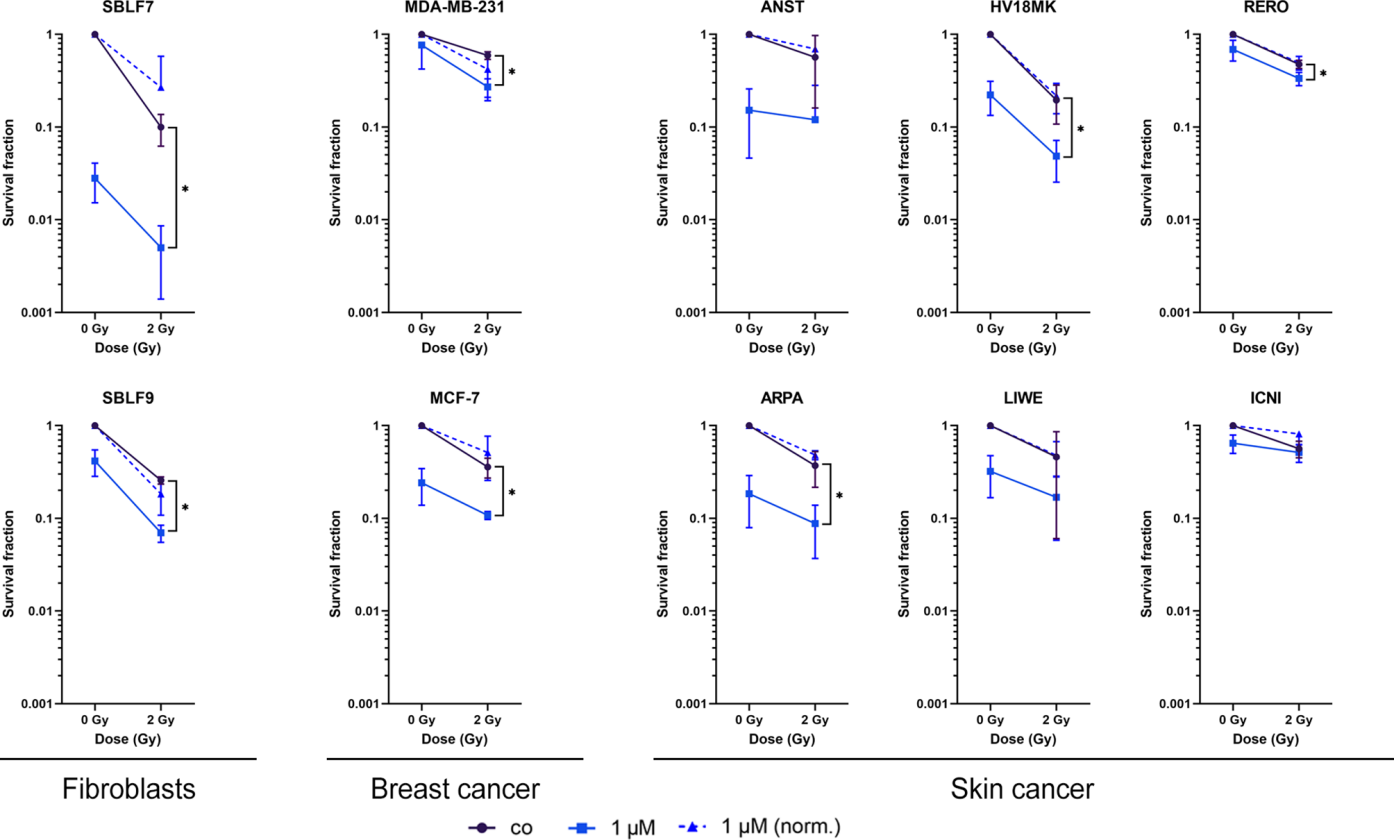
Survival fractions of healthy fibroblasts and breast cancer and skin cancer cells. Clonogenicity of two healthy fibroblasts and two breast cancer and six skin cancer cell lines. Cells were treated with 1 µM palbociclib w/o 2 Gy. Values were normalized to the irradiated control fraction (blue dashed line). Each value represents mean ± SD (*n* = 3). Significance was determined by one-tailed Mann–Whitney *U* test *p ≤ 0.05.

As a gold standard in radiation biology, colony-forming assays were used to investigate the interaction of palbociclib and irradiation within healthy and cancer cells (**Figure 2**). Both healthy fibroblasts decreased clonogenicity in a significant manner comparing combinatory therapy to irradiation alone (*p* = 0.05) in an additive manner. SBLF7 cells showed the most dramatic fall of survival in total. Breast cancer cell lines and three of six skin cancer cell lines decreased their colony-forming ability significantly (*p* = 0.05) in an additive manner. Additionally, we normalized our data to get a better understanding of antagonistic, additive, or synergistic effects. No significance could be detected in all tested cell lines.

### Palbociclib Induces Senescence

**Figure 3:**
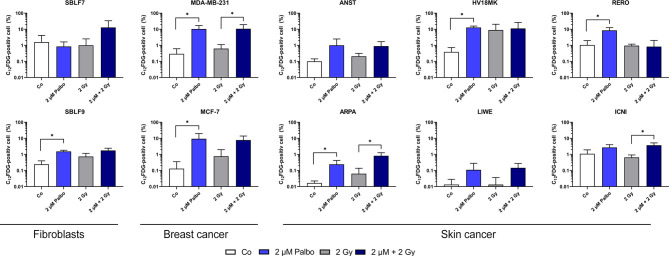
C12-FDG staining as indicator of cellular senescence. Proportion of C12-FDG-positive cells after 10 days of incubation in 10 different cell lines (healthy fibroblast SBLF7 and SBLF9, breast cancer MDA-MB-231 and MCF-7, and skin cancer ANST, ARPA, HV18MK, LIWE, RERO, and ICNI cells). Cells were treated for 24 h and irradiated with a single dose of 2 Gy. Each value represents mean ± SD (*n* = 3). Significance was determined by one-tailed Mann–Whitney *U* test *p ≤ 0.05.

Beside a wide range of effects like clonogenicity, survival, and cell death, colony-forming assay can also act as an indicator for senescence. We analyzed our malign and non-malign cell lines more deeply using a C12-FDG staining. Since senescence is a time-dependent process, we observed C12-FDG positivity initially of one healthy and one cancer cell line on days 3, 6, and 10 (**Supplementary Figure S4**). The effect was best detectable on day 10. Subsequently, all cell lines were tested at day 10 after treatment (**Figure 3**). Healthy fibroblasts did not increase C12-FDG positive cells significantly after combination treatment, but SBLF9 with monotherapy palbociclib (*p* = 0.05). One breast cancer and two out of six skin cancer cell lines raised the amount of C12-FDG positive cells at day 10 after concomitant KI + IR therapy. Noticeably, palbociclib treatment alone already raises C12-FDG positive proportion of cells in five out of eight cancer cell lines (*p* = 0.05).

Additionally, as a first step of deeper analysis of interactions and outcomes of kinase inhibitors and concomitant irradiation, we analyzed cells after 48 h of treatment. We investigated mRNA expression levels of Cyclin D1, RPTOR, Myc, SMAD3, MARCKS, RICTOR, p16, and FOXM1, which are related to CDK4/6 as, e.g., cellular inhibitor, binding partner, or central proteins of downstream pathways in our 10 cell lines including healthy fibroblasts, breast cancer, and skin cancer cells. Expression was normalized to housekeeping genes HMBS, RPL30, and UBC. Additionally, expression of treated samples was normalized to the corresponding untreated control to verify down- or upregulation plotted in the heatmaps (**Supplementary Figures S5A–H**).

Noticeable, relevant downregulation of senescence-inhibiting FOXM1 was found after combination therapy in all cell lines and in almost all sample of single therapy treatment (**Supplementary Figure S5H**). Furthermore, downregulation of p16 mRNA was found after IR in seven of eight and after combination therapy in five out of eight cancer cell lines (**Supplementary Figure S5G**). Interestingly, skin cancer cell line ANST showed overall diverse behavior, regarding downregulation of Cyclin D1, Myc, and SMAD3 as well as upregulation of MARCKS and RICTOR, compared to all other tested cell lines. Another special case seems to be skin cancer cell line LIWE, which showed downregulation after monotherapy (KI or IR) of Cyclin D1, RPTOR, and Myc in obvious contrast to the upregulation after combination of KI and IR. Overall, our mRNA expression data showed slight regulations in healthy fibroblasts compared to the wide-ranging behavior of the tested malign cancer cells. Nonetheless, these preliminary data should be held as first screening of mRNA expression and will lead to further analysis of the most interesting regulated genes of interest (GOI) in depth.

### Palbociclib Induces a Cell Cycle Block

**Figure 4:**
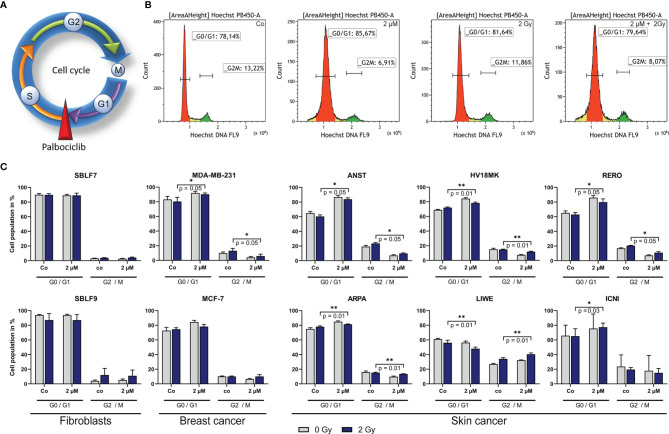
Influence of palbociclib on cell cycle of different cell lines. **(A)** Palbociclib arrest cells in the G1 phase of cell cycle by blocking the S/G1 phase checkpoint. **(B)** Representative histograms depicting the gating strategy of Hoechst staining (DNA) in breast cancer cell line MCF-7 after treatment (Co/2 µM/2 Gy/2 µM + 2 Gy). **(C)** Population of cells in the G0/G1 or G2/M phase, respectively. Cells were treated with 2 µM palbociclib, 2 Gy dose irradiation, or a combination of both. Each value represents mean ± SD (*n* = 4). Significance was determined by one-tailed Mann–Whitney *U* test *p ≤ 0.05, **p = 0.01.

In general, palbociclib binds to CDK4 and 6, which are central proteins involved in controlling progression through the G1 phase of the cell cycle (**Figures 4A, B**) (26). Furthermore, the cell cycle status of cancer cells is relevant for irradiation therapy since the G2 phase is known to be more prone to IR (27). In healthy fibroblasts, no changes were detectable treating cells with palbociclib and irradiation (**Figure 4C**). In one of both breast cancer cell lines and four out of six skin cancer cell lines, the cell population in the G2/M phase decreased significantly after combination treatment compared to IR alone (ANST, RERO: *p* = 0.05; HV18MK, ARPA: *p* = 0.01), whereas the G0/G1 phase increased. Unexpectedly, we found in the skin cancer cell line LIWE an increase of cells in the G2/M phase of cell cycle (*p* = 0.01). To study if this cell cycle block is transient or durable, the effect on markers of cellular senescence was studied.

## Discussion

Flow cytometric analysis of cell death in cancer and healthy tissue cell lines indicated tendencies regarding that palbociclib influences apoptosis and necrosis in a cell line-specific manner but rarely significant. Healthy cells seem to be less affected than tumor cells, but we assume that cell death is not the main mechanism of action of the CKD4/6 inhibitor palbociclib. The obvious intercellular differences might be explained by a heterogeneous mutation profile of our different cell lines. According to the distribution of cancer patients into different entities and subtypes (e.g., breast and lung cancer), it is known that every patient shows different mutations in depth. Our patient-derived skin cancer cell lines, which are harboring a more primary character, are used to represent a wider range of patient-specific mutations. Additionally, there is evidence that status of p53 and DNA damage repair proteins like ATM influences the outcome of palbociclib treatment in the context of irradiation (14).

Palbociclib plays an important role in cell cycle regulation as inhibitor of CDK4 and CDK6. Since the distribution of cell cycle phases is of relevant matter in radiation biology, we investigated the distribution of cell in the G0/G1 and G2/M phase. The G2 phase is known to be more sensitive to radiation (27). Healthy fibroblasts, representing normal tissue, were not affected by palbociclib significantly. Cancer cells responded controversially, as four out of six cell lines decreased G2/M populations. Interestingly, we also found one cell line that increased population of cells in the G2/M phase. This might be beneficial for radiosensitivity regarding improved local tumor control. However, our data suggest that blocking the cell cycle permanently could lead to senescence as the main mechanism of palbociclib treatment.

Regarding the results of our colony-forming assays, clear evidence is found for the higher effect of palbociclib on clonogenicity, survival, and senescence, since palbociclib blocks the cell cycle, but does not lead to cell death (28). Senescence can be triggered by, e.g., cellular stress like DNA damage or aging processes and could lead to irreversible growth arrest called stress-induced or replicative senescence, respectively (29). A senescent phenotype is also relevant in cancer and overexpression of senescence markers can be found in malign cells and tissues (30). Noticeable, colony forming was strongest decreased in healthy control cell line SBLF7. Nevertheless, all malign cell lines showed clear trends to lower cell survival rates after combination therapy compared to irradiation alone. Since irradiation is known to induce senescence as well, the combination therapy was assumed to increase this non-proliferating phenotype more efficiently (31). Additionally, effective combination of palbociclib and IR was previously described in glioblastoma multiforme intracranial xenografts (32). In our setting, we did not detect synergistic effects of simultaneous KI and IR therapy on senescence. However, this study leads us to the assumption that tumor control can be improved by combination therapy in an additive manner. To study the ability of palbociclib to induce senescence, further analysis of C12-FDG status was performed. Palbociclib clearly induced senescence in breast cancer cells. The melanoma cell lines responded very diversely. This probably indicates the inter-individual differences between every single cancer patient, according to our cell death analysis.

The preliminary analysis of mRNA expression under kinase inhibitor, irradiation, or combination therapy showed again a wide range of effects in different cancer cell lines. Eight CDK4/6 related targets were chosen to verify different ways of action of CDK4/6 blocking including, e.g., autophagy, apoptosis, migration, and senescence (33). FOXM1 was downregulated in all cell lines after combination therapy. Strong downregulation correlated with a significant increase of C12-FDG positive cells after combination therapy in, e.g., ARPA and ICNI cells. Unfortunately, for the unexpected behavior regarding upregulation of FOXM1 after palbociclib compared to significant enhancement of C12-FDG in HV18MK and RERO, we lack an appropriate explanation. Further analysis of the mutational profile of our patient-derived cell lines could help to overcome this point. Palbociclib does not lead to cell death itself, but induces senescence while blocking the cell cycle in G0/G1. As FOXM1 is known to inhibit senescence (33, 34) and, more importantly, is known to be reduced throughout palbociclib treatment (35), the decreased expression supports this idea. Unexpectedly, even after IR, downregulation of FOXM1 could be detected, an effect Li et al. (2019) published as a mechanism of pulmonary fibrosis (36). Additionally, the expression of p16, which is known as intracellular CDK4 and CDK6 inhibitor, was downregulated in five out of eight cancer cell lines after combination treatment, but might be triggered mainly by IR. Blocking the cell cycle with palbociclib may lead to less CKD4/6 and less p16 expression as a feedback loop (37). Overall, treatment with palbociclib or a combination with irradiation seems to induce senescence, but not cell death. Cellular processes like migration or autophagy may be involved, but further analyses will be necessary to understand how palbociclib influences these pathways.

Taken together, our data give evidence of inducing senescence as a main mechanism of palbociclib. However, there are some limitations, which should be considered for further research. Using patient-derived cell lines enables us to more real-life models than commercially available cell lines, which are long-term adapted to flasks and incubators. Characterization of the mutation profile of standard tumor suppressor and oncogenes might be a future strategy to explain the diverse response. Particularly, the p53 status should always be analyzed in future analyses focusing on cellular mechanisms of palbociclib. Melanoma show a high frequency of mutations in the CDK4 pathway and CDK4/6 inhibitors have been beneficial in breaking resistance (38). Thus, CDK4/6 inhibitors are potential candidates for therapy of melanoma especially as our data show in combination with RT.

## Conclusion

Palbociclib induces cellular senescence in healthy skin fibroblasts, breast cancer, and melanoma cells. Cell death is only a secondary mechanism of action of palbociclib treatment. Concomitant RT leads to an increased cellular growth arrest in an additive manner. Since CDK4/6 is known to promote cancer progression in many entities (13–16), palbociclib has been approved for therapy of breast cancer. Its efficacy should also be studied in combination with RT.

## Data Availability Statement

The original contributions presented in the study are included in the article/**Supplementary Material**. Further inquiries can be directed to the corresponding author.

## Ethics Statement

The studies involving human participants were reviewed and approved by Ethik-Kommission der Friedrich-Alexander-Universität Erlangen-Nürnberg (Approval No. 204_17 Bc). The patients/participants provided their written informed consent to participate in this study.

## Author Contributions

TJ, MH, and LD contributed to conception and design of the study. TJ performed all analysis. TJ organized the database. TJ performed the statistical analysis. TJ wrote the first draft of the manuscript. RF, LH, and LD provided the resources. All authors contributed to manuscript revision, read, and approved the submitted version.

## Conflict of Interest

MH reports collaborations outside this project with Merck Serono (advisory role, speakers’ bureau, honoraria, travel expenses, and research funding); MSD (advisory role, speakers’ bureau, honoraria, travel expenses, research funding); AstraZeneca (research funding); Novartis (research funding); BMS (advisory role, honoraria, speakers’ bureau); and Teva (travel expenses).

The remaining authors declare that the research was conducted in the absence of any commercial or financial relationships that could be construed as a potential conflict of interest.

## Publisher’s Note

All claims expressed in this article are solely those of the authors and do not necessarily represent those of their affiliated organizations, or those of the publisher, the editors and the reviewers. Any product that may be evaluated in this article, or claim that may be made by its manufacturer, is not guaranteed or endorsed by the publisher.

## Abbreviations

7-AAD, 7-amino-actinomycin D; APC, Allophycocyanin; AUC, Area under curve; BSA, Bovine serum albumin; CDK, Cyclin-dependent kinase; CNS, Central nervous system; Co, Control; DAPI, 4′,6-Diamidin-2-phenylindol; DMSO, Dimethyl sulfoxide; DNA, Deoxyribonucleic acid; EMA, European Medicines Agency; ER, Estrogen receptor; FACS, Fluorescence-associated cell sorting; FBS, Fetal bovine serum; FDA, Food and Drug Administration; KI, Kinase inhibitor; IF, Immune fluorescence; IR, Irradiation; NEA, Non-essential amino acid; PBS, Phosphate-buffered saline; PR, Progesterone receptor; qPCR, Quantitative Polymerase chain reaction; Rb, Retinoblastoma protein; SD, Standard deviation; sf, Survival fraction; TBS, Tris-buffered saline; TNBC, Triple-negative breast cancer.
