# Supplementary material for: Palbociclib Induces Senescence in Melanoma and Breast Cancer Cells and Leads to Additive Growth Arrest in Combination With Irradiation
Source: Front Oncol. 2021 Oct 13;11:740002. doi: 10.3389/fonc.2021.740002 (PMC8548830; doi:10.3389/fonc.2021.740002)
Supplement: Supplementary file 1 [file DataSheet_1.pdf]

Supplementary Material

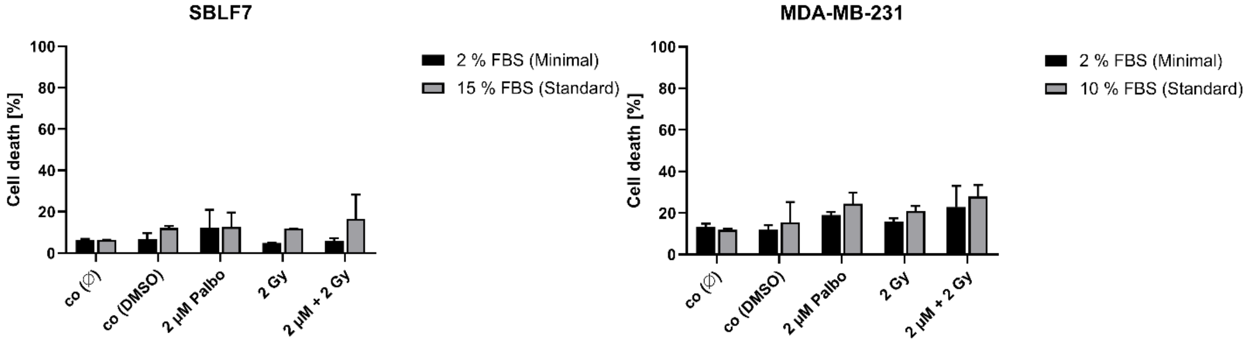

Supplementary Figure S1: Cell death analysis of healthy tissue SBLF7 cells and breast cancer cell line MDA-MB-231 under starved (2 %) and standard (10 % / 15 %) FBS concentration. Cells were treated with 2 µM Palbociclib (dissolved in DMSO), 2 Gy irradiation or a combination of both. Additionally, one control without and one including similar amount of DMSO (according to Palbociclib treatment) were used. Each value represents mean ± SD (n = 2).

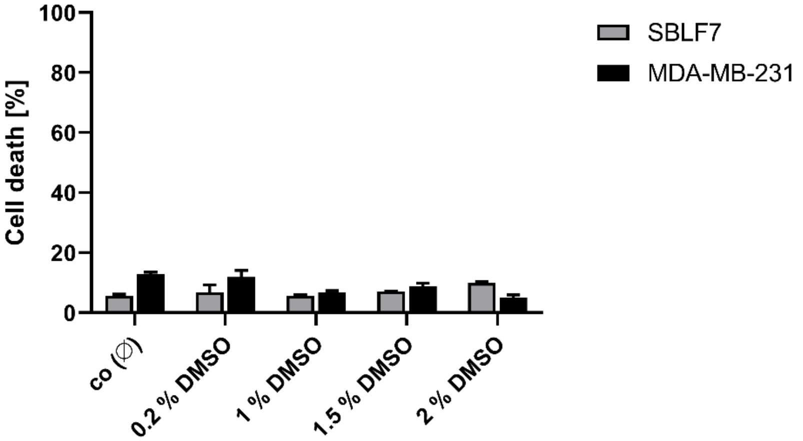

Supplementary Figure S2: Cell death analysis in healthy tissue SBLF7 cells and breast cancer cell line MDA-MB-231 under different concentration of DMSO. Each value represents mean ± SD (n = 2).

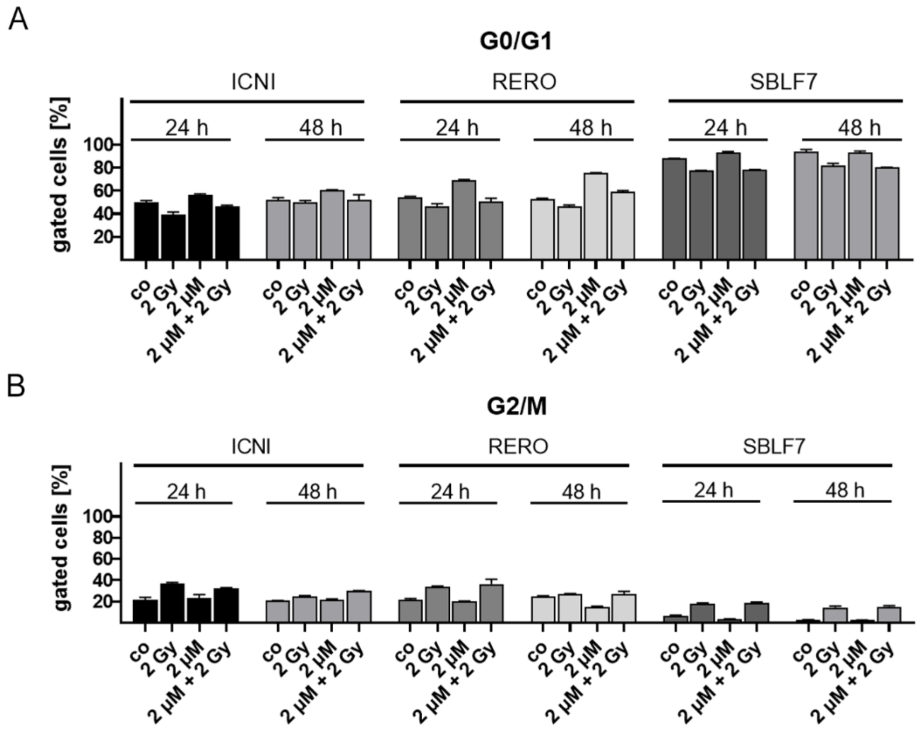

Supplementary Figure S3: Analysis of cell cycle after 24 h and 48 h of treatment with IR, palbociclib or a combination of both in skin cancer cells and healthy fibroblasts. Two different malign melanoma cells (ICNI, RERO) as well as healthy control cells (SBLF7) were treated for 24 or 48 hours. Cell cycle distribution was measured using Hoechst staining (33342, Molecular probes) via flow cytometry (Cytoflex, Beckman coulter). A decrease of G0/G1 after 2 Gy IR was detectable, but just slight differences between 24 h and 48 h. Each value represents mean ± SD (n = 2).

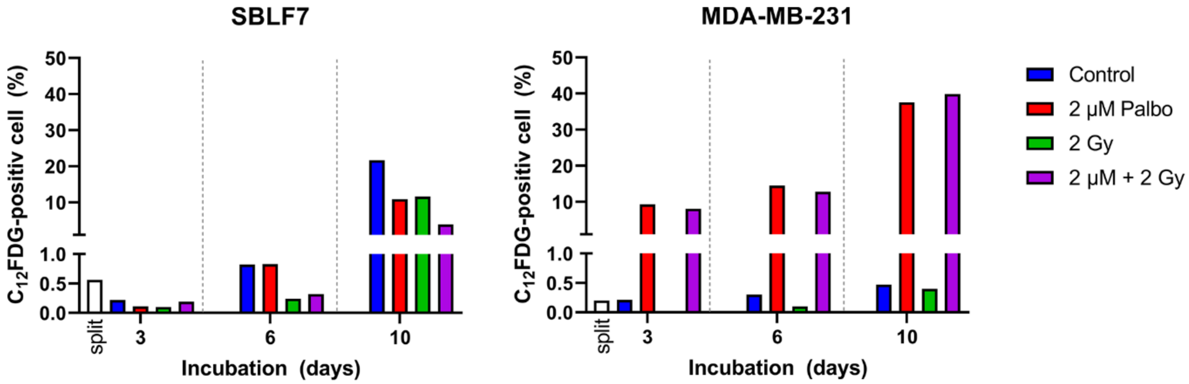

Supplementary Figure S4. Time-lapse C12-FDG staining as indicator of cellular senescence on day 3, 6 and 10. Development of C12-FDG positive cells after 3, 6 and 10 days of incubation in healthy fibroblasts SBLF7 and breast cancer cell line MDA-MB-231. Cells were treated for 24 h and irradiated

with a single dose of 2 Gy. Each value was measured once to find best incubation period to investigate senescence via quantity of C12-FDG positive cells.

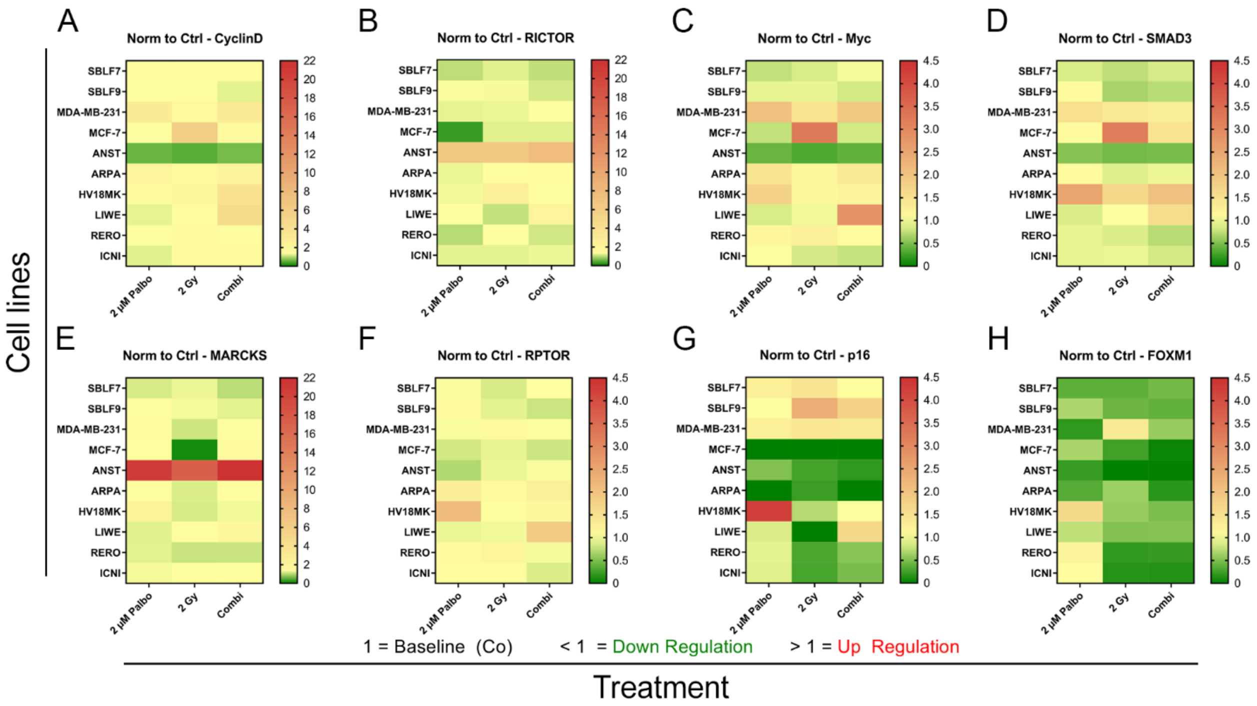

Supplementary Figure S5: Heatmaps of the mRNA expression analysis (qPCR) of eight target genes in ten cell lines. Foldchange of mRNA expression level of Cyclin D1, RPTOR, Myc, SMAD3, MARCKS, RICTOR, p16 and FOXM1 was tested after 48 h of treatment in 10 different cell lines (healthy fibroblast SBLF7 and SBLF9, breast cancer MDA-MB-231 and MCF-7 and skin cancer ANST, ARPA, HV18MK, LIWE, RERO and ICNI cells). Data was normalized to untreated control and housekeeping genes HMBS, RPL30 and UBC. Assay was performed in technical duplicates of one biological sample. Heatmaps show down regulation (green) or up regulation (red) of analyzed target genes. Two technical replicates (duplicate wells) from one RNA/cDNA preparation (one biological sample) were measured (n = 1).
